# Post Hoc Analysis of Frailty and Tracheostomy Risk in Older Patients Intubated and in an Intensive Care Unit in Japan: An Inverse Association in Older Patients with Advanced Age

**DOI:** 10.31662/jmaj.2025-0455

**Published:** 2026-03-19

**Authors:** Takehide Umeda, Takashi Hongo, Mototaka Inaba, Hiromichi Naito, Yoshinori Ariyasu, Takashi Yorifuji

**Affiliations:** 1 Department of Epidemiology, Graduate School of Medicine, Dentistry and Pharmaceutical Sciences, Okayama University, Okayama, Japan; 2 Emergency and Critical Care Center, Kurashiki Central Hospital, Kurashiki, Japan; 3 Department of Emergency, Critical Care, and Disaster Medicine, Okayama University Graduate School of Medicine, Dentistry, and Pharmaceutical Sciences, Okayama, Japan; 4 Department of Clinical Engineering, National Hospital Organization Shikoku Cancer Center, Matsuyama, Japan

**Keywords:** frailty, respiration, tracheostomy, critical care outcomes, aged

## Abstract

**Introduction:**

This post hoc analysis of a prospectively collected intensive care unit (ICU) cohort examined the association between frailty and the likelihood of tracheostomy in older Japanese patients (aged ≥65 years). Frailty, a condition of increased vulnerability to stressors, is common among older patients in the ICU and may influence clinical decision-making and outcomes. We aimed to explore whether baseline frailty is associated with the likelihood of receiving tracheostomy in older patients in the ICUs.

**Methods:**

We analyzed data from a multicenter prospective study conducted from November 2019 to April 2020 at 17 hospitals in Japan. Patients aged ≥65 years, admitted directly from the emergency department, and requiring mechanical ventilation, were included. After excluding early deaths (≤5 days) or treatment-limit cases, 363 patients with intubation remained. Frailty was assessed using the Clinical Frailty Scale (CFS), with primary cutoff CFS ≥4. The primary outcome was the occurrence of tracheostomy during ICU stay. Risk ratios (RRs) and 95% confidence intervals (CIs) were estimated using generalized linear models with a Poisson distribution, adjusted for age, sex, Charlson Comorbidity Index, and Acute Physiology and Chronic Health Evaluation II score.

**Results:**

Among 363 patients, patients with frailty (CFS ≥4) had a significantly lower adjusted risk of having tracheostomy than did those with less frailty (CFS <4) (adjusted RR: 0.40; 95% CI: 0.27-0.61). In patients aged ≥75 years, the adjusted RR for CFS ≥4 was 0.32 (95% CI: 0.20-0.50), indicating a pronounced reduction in tracheostomy use among patients with frailty.

**Conclusions:**

Frailty (CFS ≥4) was independently associated with a lower likelihood of tracheostomy, particularly in patients aged ≥75 years.

## Introduction

Frailty is a clinically recognized condition characterized by increased vulnerability in older patients. It is characterized by increased vulnerability to stressors that lead to functional impairment and health disadvantages ^(1)^. Previous studies have shown that frailty is associated with increased hospital mortality, complications, and prolonged hospital stay ^(2), (3), (4)^. Recently, the number of older patients admitted to intensive care units (ICU) has increased; thus, the impact of frailty on outcomes in older patients in the ICU has received increasing attention ^(5)^.

Although the findings are limited and inconsistent, some studies have indicated the negative impact of frailty on outcomes, especially related to extubation or tracheostomy, in the ICU. In a prior study, frailty was independently associated with an extended duration of mechanical ventilation and extubation failure ^(6), (7)^. This result is reasonable because frailty and related muscle loss (i.e., sarcopenia) can hinder deep breathing and the ability to clear secretions, complicating the weaning process from ventilators for patients with frailty in the ICU ^(8)^. In a previous study, Fernando et al. ^(7)^ reported that frailty was associated with extubation failure and marginally increased the odds of tracheostomy (adjusted odds ratio 1.17 [95% confidence intervals (CI): 1.01-1.36]) among patients mechanically ventilated in the ICU. However, their analysis included a wide age range, starting from 18 years, and was conducted in a Western context. It remains unclear whether the same association holds true in older populations, particularly in super-aged societies such as Japan. No prior study has specifically focused on the relationship between frailty and tracheostomy in older patients intubated in the ICU in this setting, leaving a gap in understanding how frailty influences procedural decisions in geriatric critical care medicine. The decision to perform tracheostomy involves a complex evaluation of long-term prognosis, comorbidities, age, patient preferences, and overall goals of care ^(9)^. Consequently, patients with marked frailty may opt out of tracheostomy procedures, even when such interventions are clinically advisable, on the basis of their personal preferences. This decision may change with each medical setting and the older population, especially in the advanced older population.

This study aimed to characterize tracheostomy practices among patients intubated in the ICU, aged ≥65 years in Japan, a super-aged society, and to explore whether baseline frailty is associated with the likelihood of tracheostomy in this population.

## Materials and Methods

### Study area and participants

This study is a post hoc analysis of a prospective, multi-center observational study conducted across 17 hospitals in Japan (UMIN000037430), originally designed to evaluate the association between frailty and 6-month mortality in critically ill older patients. The original cohort included patients aged ≥65 years who were admitted to the ICU directly from the emergency department (including inter-facility transfers) and had baseline Clinical Frailty Scale (CFS) scores recorded within 24 h of ICU admission. Patients were excluded from the original study if they or their surrogates declined participation or if baseline CFS assessment was not possible. For this post hoc analysis, we focused on patients intubated in the ICU. Patients who died within 5 days of ICU admission were excluded. Patients who died in the ICU with documented limitations on life-sustaining treatment (Do Not Attempt Resuscitation orders, withholding of escalation, or planned withdrawal of care) were also excluded, given tracheostomy was typically not considered in these end-of-life cases. These exclusions were applied to reduce competing risk bias that could affect the evaluation of tracheostomy decisions. The main analysis included 363 patients: survivors in ICU both with and without treatment limitations, in addition to non-survivors in ICU without treatment limitations. The detailed participant flow and cohort counts are presented in the Results section. The sample size of this post hoc analysis was defined by the number of eligible intubated cases in the original cohort.

Patient enrollment occurred over four consecutive months at each participating center between November 2019 and April 2020. The inclusion criteria were admission to the ICU directly from the emergency department (including inter-facility transfers) and age ≥65 years at the time of ICU admission. ICU admission decisions were made by emergency physicians, and eligible patients were screened consecutively by the attending staff. On ICU admission, baseline CFS scores were collected, along with patient characteristics such as age, sex, height, weight, Charlson Comorbidity Index (CCI), illness origin, and Acute Physiology and Chronic Health Evaluation II (APACHE II) score, which was used to assess the severity of the acute illness. Information on mechanical ventilation and life-sustaining treatment was also recorded. CCI and APACHE II scores were used to quantify chronic comorbidity and acute illness burden, respectively.

### Primary outcome

The primary outcome of this study was the occurrence of a new tracheostomy during the ICU stay, defined as any tracheostomy procedure performed between ICU admission and ICU discharge (including discharge due to death), as documented in the procedure records or electronic medical records.

### Frailty

Frailty was assessed using the CFS, a 9-point tool developed by Rockwood et al. ^(10)^ that categorizes frailty from 1 (very fit) to 9 (terminally ill). The CFS was chosen because it is a validated and widely used tool for assessing frailty in critically ill older adults. It is simple to administer in acute settings and correlates well with short- and long-term prognosis ^(2)^. A validated Japanese-translated version was used in this study. Baseline CFS scores were determined through interviews with patients or surrogates, referring to the patient’s status approximately two weeks before the acute illness. Scores were recorded by attending physicians or nurses immediately after the ICU admission. In our study, frailty was defined using a primary cutoff of CFS ≥4. Sensitivity analyses were conducted using an alternative cutoff of CFS ≥5.

### Statistical analyses

Descriptive statistics were used to summarize the demographic characteristics of the patients. Continuous variables were summarized using mean ± standard deviation for approximately symmetric distributions (e.g., age) in the main cohort and median with interquartile range for skewed distributions (e.g., APACHE II, CCI), based on visual assessment and standard practice in critical care research. Frequencies and percentages were calculated for categorical variables, such as sex and CFS scores. This descriptive analysis compared frailty distributions across patient groups before further statistical testing.

Risk ratios (RRs) and 95% CI were estimated using generalized linear models with a Poisson distribution and robust standard errors, an established approach for analyzing binary outcomes in cohort studies. The RRs were calculated by comparing the incidence of tracheostomy in patients with a CFS score of ≥4 and those with a score of <4. After estimating the crude RRs, we adjusted for potential confounders, including age, sex, CCI, and APACHE II score, to control for the effects of these variables on the outcome and provide adjusted RRs. We further stratified the analysis by age groups (≥75 and <75 years) to explore whether the impact of frailty on tracheostomy risk differed across age strata, recognizing that age may modify the effect of frailty.

All statistical analyses were performed using STATA version 19.5 (Stata Corp LP, College Station, TX, USA). This post hoc analysis was approved by the Ethics Committee of the Graduate School of Medicine, Dentistry and Pharmaceutical Sciences, Okayama University, and Okayama University Hospital (number 2412-051).

## Results

**Figure 1.**
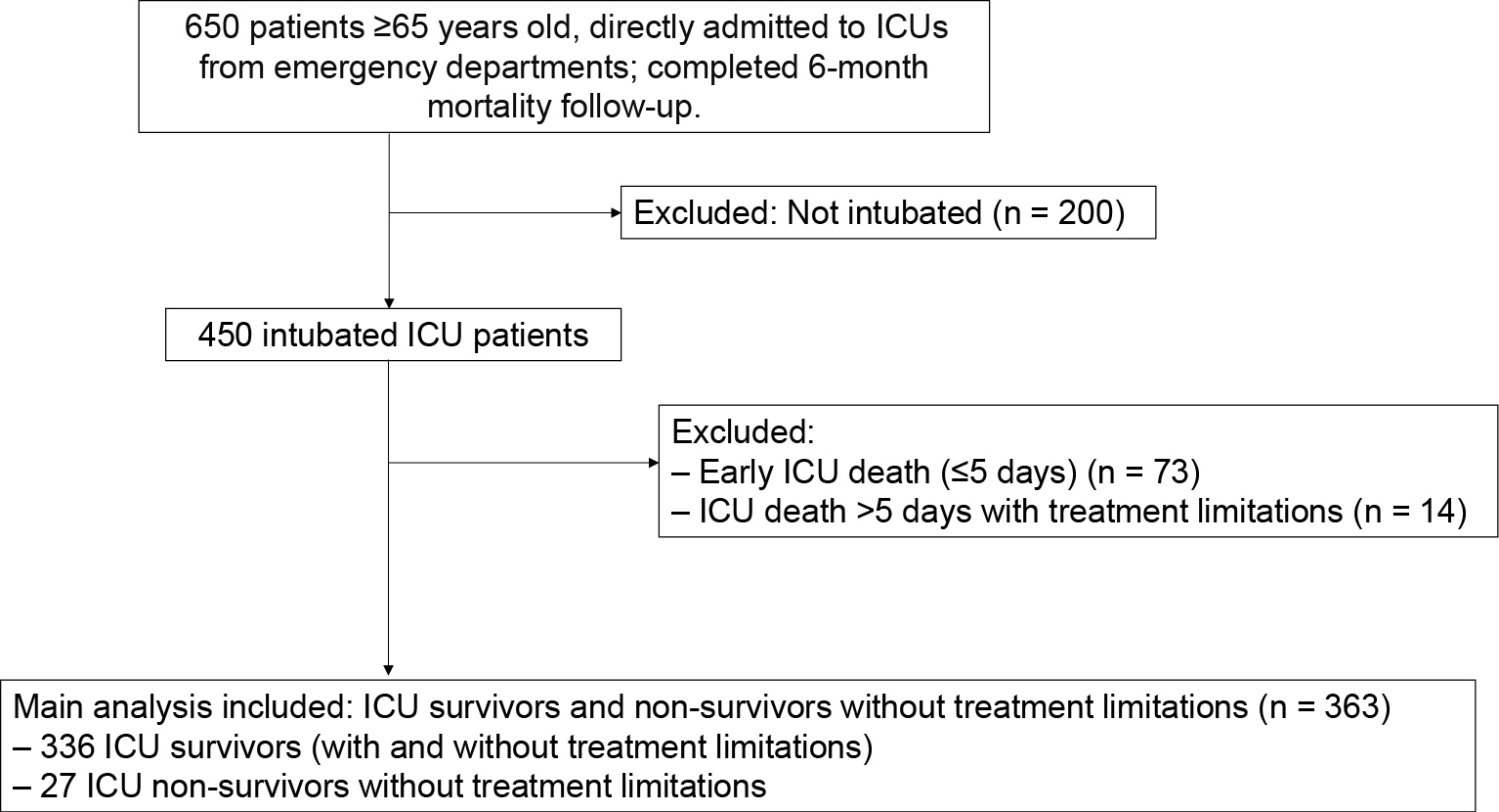
Flowchart detailing the study participants. ICU, intensive care unit.

Of the 650 patients enrolled in the original prospective cohort, 450 were intubated and considered for post hoc analysis. Among them, 73 patients who died early in the ICU (≤5 days) and 14 who died later in the ICU with documented treatment limitations were excluded from the study. The main analysis included 363 patients: 336 survivors in ICU and 27 non-survivors in ICU without life-sustaining treatment limitations (Figure 1).

Baseline characteristics, including comorbidities and illness categories, are summarized in Table 1. Among patients without life-sustaining treatment limitations (n = 297), those who underwent tracheostomy (n = 70) were older (mean age, 84.4 vs. 78.1 years), more frequently male (87.2% vs. 64.3%), and had longer ICU stays (median, 13 vs. 4 days) than those who did not undergo tracheostomy (n = 227). The tracheostomy group also had a higher prevalence of chronic pulmonary disease (32.9%) and tended to show less frailty, with 45.7% scoring CFS 3 and most scoring CFS ≤3. In contrast, among survivors in ICU with treatment limitations (n = 66), the 24 patients with tracheostomy were markedly older (mean age, 90.1 years), with extremely high rates of chronic pulmonary disease (91.7%) and malignancy (83.3%). These patients also showed severe frailty: 91.7% had a CFS score of 6, and none had a CFS score below 4. Their ICU stays were similarly prolonged (median, 15 days). The 42 patients without tracheostomy with treatment limitations showed more variability in CFS scores but still exhibited high overall frailty, with 57.1% scoring CFS 3 and 14.3% scoring CFS 7.

**Table 1.** Baseline Characteristics of Patients Intubated in the ICU with and without Life-Sustaining Treatment Limitation, Stratified by Survival, and Tracheostomy Status.

	Survivors and non-survivors without life-sustaining treatment limitation^a^		Survivors with life-sustaining treatment limitation	
	Non-tracheostomy (n = 227)	Tracheostomy (n = 70)	Non-tracheostomy (n = 42)	Tracheostomy (n = 24)
Individual-level characteristics				
Mean age (year) ± SD	78.1±6.8	84.4±8.8	81.1±5.7	90.1±4.7
Sex, male, n (%)	146 (64.3)	41 (87.2)	33 (78.6)	1 (4.2)
CCI, median (IQR)	4 (3-6)	5 (5-7)	6 (5-6)	8 (7-8)
APACHE, II score, median (IQR)	24 (13-31)	23 (15-33)	27 (22-32)	27 (22-36)
ICU admission category^b^, n (%)				
Cardiology	41 (18.1)	23 (32.9)	4 (9.5)	0 (0)
Pulmonary	37 (16.3)	27 (28.6)	7 (16.7)	3 (12.5)
Gastrointestinal	46 (20.3)	1 (1.4)	1 (2.4)	0 (0)
Neurology	65 (28.6)	7 (10)	24 (57.1)	20 (83.3)
Trauma	19 (8.4)	11 (15.7)	0 (0)	0 (0)
Endocrine	7 (3.1)	0 (0)	3 (7.1)	0 (0)
Skin/tissue	2 (0.9)	0 (0)	0 (0)	0 (0)
Urology	2 (0.9)	0 (0)	0 (0)	0 (0)
Others	8 (3.5)	1 (1.4)	3 (7.1)	1 (4.2)
ICU length of stay (days), median (IQR)	4 (3-9)	13 (8-15)	3 (3-9)	15 (11-15)
Major comorbidities, n (%)				
Dementia	69 (30.4)	27 (38.6)	12 (28.7)	2 (8.3)
Congestive heart failure	30 (13.2)	2 (2.9)	1 (2.4)	1 (4.2)
Chronic pulmonary disease	14 (6.2)	23 (32.9)	4 (9.5)	22 (91.7)
Malignancy	17 (7.5)	5 (7.1)	3 (7.1)	20 (83.3)
^c^CFS score, n (%)				
1	19 (8.4)	5 (7.1)	0 (0)	0 (0)
2	56 (24.7)	21 (30)	3 (7.1)	0 (0)
3	34 (15)	32 (45.7)	24 (57.1)	0 (0)
4	52 (22.9)	5 (7.1)	3 (7.1)	1 (4.2)
5	32 (14.1)	1 (1.4)	0 (0)	1 (4.2)
6	7 (3.1)	3 (4.3)	4 (9.5)	22 (91.7)
7	26 (11.5)	2 (2.9)	6 (14.3)	0 (0)
8	1 (0.4)	1 (1.4)	2 (4.8)	0 (0)

APACHEⅡ: Acute Physiology and Chronic Health Evaluation II; CCI: Charlson Comorbidity Index; CFS: Clinical Frailty Score; SD: standard deviation. ^a^excluded early ICU death ≤5 days. ^b^Nine subjective categories of illness etiology were defined by the research group, and one was selected by the attending physician for each patient. ^c^No patient was scored as CFS 9.

Table 2 summarizes the association between frailty and tracheostomy among 363 patients intubated in the ICU, including survivors in ICU with and without treatment limitations and non-survivors in ICU without treatment limitations. When using a cutoff of CFS ≥4 to define frailty, 21.3% of patients with frailty (36/169) underwent tracheostomy, compared with 29.9% of patients without frailty (58/194). In the crude analysis, frailty defined by CFS ≥4 was associated with a lower risk of tracheostomy (RR 0.71; 95% CI: 0.50-1.02), although this was not statistically significant. After adjusting for age, sex, CCI, and APACHE II score, frailty (CFS ≥4) was significantly associated with a reduced likelihood of tracheostomy (adjusted RR, 0.40; 95% CI: 0.27-0.61).

**Table 2.** Risk of Tracheostomy by Clinically Evident Frailty in the Survivors Intubated in the ICU both with and without Treatment Limitations, in addition to Non-Survivors in ICU without Treatment Limitations (n = 363)^a^ in Different Cutoffs of CFS (CFS4 and CFS 5).

	Tracheostomy case/n (%)	Risk ratio (95% CI) for tracheostomy	
		Crude	Adjusted^b^
CFS score ≥4	36/169 (21.3%)	0.71 (0.5-1.02)	0.4 (0.27-0.61)
CFS score <4	58/194 (29.9%)	Reference	Reference
			
CFS score ≥5	30/108 (27.8%)	1.11 (0.76-1.6)	0.49 (0.31-0.79)
CFS score <5	64/255 (25.1%)	Reference	Reference

CFS: Clinical Frailty Scale; CI: confidence interval. ^a^Excluded early ICU death ≤5 days. ^b^Adjusted for age, sex, Charlson Comorbidity Index, and APACHE II score.

Sensitivity analysis using CFS ≥5 produced similar results, supporting the robustness of our findings. When using a stricter cutoff of CFS ≥5, 27.8% of patients with frailty (30/108) underwent tracheostomy compared with 25.1% of patients without frailty (64/255). The crude analysis did not show a clear association (RR, 1.11; 95% CI: 0.76-1.60). However, after adjustment, CFS ≥5 was significantly associated with a reduced likelihood of tracheostomy (adjusted RR 0.49, 95% CI: 0.31-0.79).

These findings suggest that frailty, when appropriately adjusted for confounders, was associated with a lower probability of receiving tracheostomy, regardless of whether CFS ≥4 or ≥5 was used as the cut-off value.

Table 3 summarizes the risk of tracheostomy by clinically evident frailty in survivors intubated in ICU both with and without treatment limitations, in addition to non-survivors in ICU without treatment limitations, stratified by age group using different frailty cutoffs (CFS ≥4 and CFS ≥5). Among patients aged ≥75 years (n=293), frailty, defined as CFS ≥4, was associated with a significantly lower likelihood of receiving tracheostomy than in those with CFS <4. The adjusted RR was 0.32 (95% CI: 0.20-0.50), and the crude RR was 0.62 (95% CI: 0.43-0.90). Similarly, using the CFS ≥5 cutoff, patients with frailty also had a lower adjusted risk of tracheostomy (adjusted RR: 0.39; 95% CI: 0.23-0.67), with a crude RR of 0.98 (95% CI: 0.67-1.43). These age-stratified findings indicate that the inverse association between frailty and tracheostomy is concentrated in patients with advanced age.

**Table 3.** Risk of Tracheostomy by Clinically Evident Frailty in the Survivors Intubated in the ICU both with and without Treatment Limitations, in addition to Non-Survivors in the ICU without Treatment Limitations, Stratified by Age Group in Different Frailty Cutoffs (CFS4 and CFS 5)^a^.

		Tracheostomy case/n (%)^b^	Risk ratio (95% CI) for tracheostomy	
			Crude	Adjusted^c^
Age ≥75	CFS score ≥4	33/147 (22.4%)	0.62 (0.43-0.9)	0.32 (0.2-0.5)
Age ≥75	CFS score <4	53/146 (36.3%)	Reference	Reference
				
Age <75	CFS score ≥4	3/22 (13.6%)	1.31 (0.34-5.05)	
Age <75	CFS score <4	5/48 (10.4%)	Reference	
				
Age ≥75	CFS score ≥5	57/193 (29.5%)	0.98 (0.67-1.43)	0.39 (0.23-0.67)
Age ≥75	CFS score <5	29/100 (29.0%)	Reference	Reference
				
Age <75	CFS score ≥5	1/8 (12.5%)	1.11 (0.15-7.99)	
Age <75	CFS score <5	7/62 (11.3%)	Reference	

APACHEⅡ: Acute Physiology and Chronic Health Evaluation II; CFS, Clinical Frailty Scale; CI: confidence interval; ICU: intensive care unit. ^a^Excluded early ICU death ≤5 days. ^b^n = 293 for age ≥75, n = 70 for age <75. ^c^Adjusted for age, sex, Charlson Comorbidity Index, and APACHE II score.

Among patients aged <75 years (n = 70), no significant association between frailty and tracheostomy was found. Using CFS ≥4, the crude RR was 1.31 (95% CI: 0.34-5.05). For CFS ≥5, the crude RR was 1.11 (95% CI: 0.15-7.99). Adjusted analyses were not performed in this subgroup because of the small number of events, to avoid model overfitting.

In Supplementary Figure 1, adjusted RRs for tracheostomy are shown according to frailty cutoffs (CFS ≥4 and ≥5) stratified by age group. Among patients aged ≥75 years, frailty was significantly associated with a lower likelihood of tracheostomy. In Supplementary Figure 1, the subgroup aged <75 years was excluded owing to small sample size and lack of adjusted estimates.

## Discussion

To the best of our knowledge, this is the first multicenter study in a super-aged society to show that frailty is inversely associated with tracheostomy likelihood among older patients in ICU, particularly among those aged ≥75 years. In this subgroup, patients with clinically evident frailty were significantly less likely to undergo tracheostomy than those without. This finding is consistent with current discussions on the appropriateness of aggressive interventions in older patients with frailty ^(8), (11)^. However, no such association was observed in older patients aged <75 years. Collectively, these findings reflect real-world clinical decision-making, in which the use of invasive procedures, such as tracheostomy, is often weighed against perceived recovery potential, long-term functional outcomes, and patient preferences, especially in the context of advanced age and frailty ^(12)^.

Previous research has indicated that patients with frailty aged ≥18 years in the general ICU population were more likely to experience extubation failure and had a higher likelihood of receiving tracheostomy than did their counterparts without frailty ^(7)^. Our findings challenge the traditional views on managing patients with frailty in the ICU by showing that patients with advanced age ≥75 years may undergo fewer tracheostomies, even though frailty is often linked with overall poorer outcomes ^(2), (12), (13)^. One possible explanation for this inverse association is that clinicians may consciously avoid tracheostomy in older patients with frailty owing to concerns regarding poor post-ICU functional recovery, prolonged rehabilitation, or reduced likelihood of meaningful survival. Unlike younger patients, those aged ≥75 years with evident frailty may be perceived as having reduced capacity to benefit from prolonged mechanical ventilation and the invasive nature of tracheostomy ^(14)^. In such cases, even without formal treatment limitations, the care team may lean toward earlier extubation attempts or transition to palliative care pathways based on the expected prognosis. Previous studies showing higher tracheostomy rates in patients with frailty may reflect broader and younger adult ICU populations, in whom long-term recovery potential is more variable and decision-making is less constrained by advanced age ^(7)^.

In this younger older subgroup (<75 years), no statistically significant association was observed between frailty and tracheostomy. This may be partly due to the limited sample size, which reduced the statistical power, and the greater clinical heterogeneity in this age group. Although definitive conclusions cannot be drawn, certain clinical characteristics may influence decision-making. In patients aged <75 years, frailty may reflect diverse etiologies, including early stage chronic disease or reversible postoperative decrease, and is not always associated with poor prognosis. Therefore, tracheostomy decisions in this group may depend more heavily on individual factors, such as baseline functional status, comorbidities, expected recovery, and personal or family preferences. Some individuals with frailty may be considered suitable for tracheostomy if their condition is reversible, whereas others may decline aggressive intervention on the basis of long-term quality-of-life considerations. This diversity in patient backgrounds and clinical judgment likely contributes to the absence of a clear statistical association and highlights the complexity of treatment planning beyond age or frailty.

The decision to use a CFS cutoff of ≥4 as the primary threshold for frailty was based on clinical considerations and prior research. Although some studies define frailty as CFS ≥5, there is growing evidence supporting the inclusion of individuals with a score of 4, often categorized as “vulnerable” or “living with mild frailty,” as clinically relevant. Fronczek et al. ^(15)^ suggested that CFS 4 marks the onset of functional limitations, and a scoping review by Church et al. ^(16)^ found that approximately 26% of frailty studies adopted a CFS ≥4 threshold. In this study, including individuals with CFS 4 allowed a more inclusive evaluation of early frailty and its potential impact on the decision to perform a tracheostomy. To ensure robustness, we conducted a sensitivity analysis using the more conventional CFS ≥5 cutoff.

The following findings are noted regarding the strengths of this study. This study used data collected from multiple medical facilities across Japan, enhancing the generalizability of the findings within this geographical context. The inclusion of various centers adds robustness to the data owing to the diverse patient demographics and clinical practices captured. Selection bias was minimized by excluding early deaths and treatment-limitation cases to reduce competing-risk effects. Misclassification bias in frailty assessment was mitigated through standardized interviews using the validated Japanese CFS.

Regarding the exclusion of early ICU deaths (≤5 days) and patients with treatment limitations, we focused our analysis on individuals for whom tracheostomy was a clinically realistic consideration. Tracheostomy is generally reserved for patients who remain on mechanical ventilation beyond the acute phase of critical illness, often after several days. Including patients who died early or had predetermined limitations would have biased our findings because such patients rarely reach the clinical decision point for tracheostomy ^(9)^. Although this exclusion criterion improves internal validity, it may also limit generalizability by omitting a subset of critically ill older patients with high frailty who may be underrepresented in studies of ICU interventions.

Regarding the other limitations of the study, the following findings are noted. Although data collection was prospective, the analysis itself was post hoc and retrospective, which could introduce biases related to the selection and classification of patients. Retrospective analyses are also limited by the data initially collected and may not account for all the relevant confounders. This study did not account for potential inter-center variations in clinical decision-making or treatment protocols, which may have influenced tracheostomy practices across institutions. In addition, CFS scores are often derived from histories taken by patients’ families or other caretakers, which may introduce inaccuracies in frailty evaluation, affecting the reliability of these assessments. Another limitation is the single-country focus. Although the study benefits from a multicenter approach within Japan, its findings might not be directly applicable to populations in other countries with different health care systems and cultural attitudes toward end-of-life care. Nevertheless, the absence of time-to-event data and outcome details, such as ventilator-free days or the exact timing of tracheostomy, limited our ability to apply survival models, such as the cumulative incidence function. This is an important methodological limitation of the post hoc design of our study. Moreover, data on ventilator liberation timing and whether patients were discharged from the ICU while still receiving mechanical ventilation were not available. This limits our ability to assess potential differences in the duration of ventilator dependence and post-ICU ventilator status. Another limitation of this study is that although it identifies a tendency to avoid tracheostomy in older patients with frailty, it does not evaluate how this practice influences long-term outcomes. The potential impact of this decision on factors such as survival rate, quality of life, and functional recovery warrants further investigation.

Future research should address the key gaps identified in this study. Prospective multicenter studies specifically designed to assess frailty and tracheostomy decision-making could help establish causal relationships and reduce the bias inherent in retrospective analyses. Furthermore, evaluating long-term outcomes, such as quality of life, return home, and caregiver burden, would provide a more comprehensive understanding of the real-world implications of tracheostomy in older patients with frailty in the ICU. Finally, qualitative studies exploring the perspectives of patients, families, and clinicians on the decision-making process surrounding tracheostomy would offer essential context to guide patient-centered care in this complex and value-laden clinical setting.

### Conclusions

Our findings newly indicate an age-specific pattern: among patients aged ≥75 years, frailty is associated with lower tracheostomy use, in contrast to patterns reported in broader adult ICU populations. This finding suggests a preference for less invasive management strategies, reflecting a shift toward patient-centered care in the ICU. Our study suggests that frailty may play a critical role in the management of older patients in the ICU, particularly in influencing decisions related to tracheostomy. This highlights the need for nuanced clinical guidelines that consider both age and frailty to optimize care for critically ill patients by aligning interventions with likely outcomes and patient preferences.

## Article Information

### Author Contributions

Takehide Umeda served as the corresponding author and was a major contributor to data analysis and manuscript writing. Takashi Yorifuji contributed significantly to data analysis and manuscript writing. The other authors participated in the data analysis and manuscript preparation. All authors have read and approved the final manuscript.

### Acknowledgments

We thank the ICU teams at the participating hospitals for their support in patient data collection. We also appreciate the participants and their families for their involvement in this study.

### Conflicts of Interest

None

### Approval Code Issued by the Institutional Review Board and the Name of the Institution(s) that Granted the Approval

This post hoc analysis was approved by the Ethics Committee of the Graduate School of Medicine, Dentistry and Pharmaceutical Sciences, Okayama University, and the Okayama University Hospital (number 2412-051). Ethical approval for the original study was obtained from the ethics committees of all 17 participating institutions, and informed consent was obtained from patients or their legal representatives before participation. The study was registered in the University Hospital Medical Information Network Clinical Trials Registry (UMIN-CTR) under the identifier UMIN000037430.

## Supplement

Supplementary Material Supplementary Figure 1. Adjusted risk ratios for tracheostomy according to age group and frailty cutoff.
