# Supplementary figures and images for: Post Hoc Analysis of Frailty and Tracheostomy Risk in Older Patients Intubated and in an Intensive Care Unit in Japan: An Inverse Association in Older Patients with Advanced Age
Source: JMA J. 2026 Mar 19;9(3):643–50. doi: 10.31662/jmaj.2025-0455 (PMC13246253; doi:10.31662/jmaj.2025-0455)

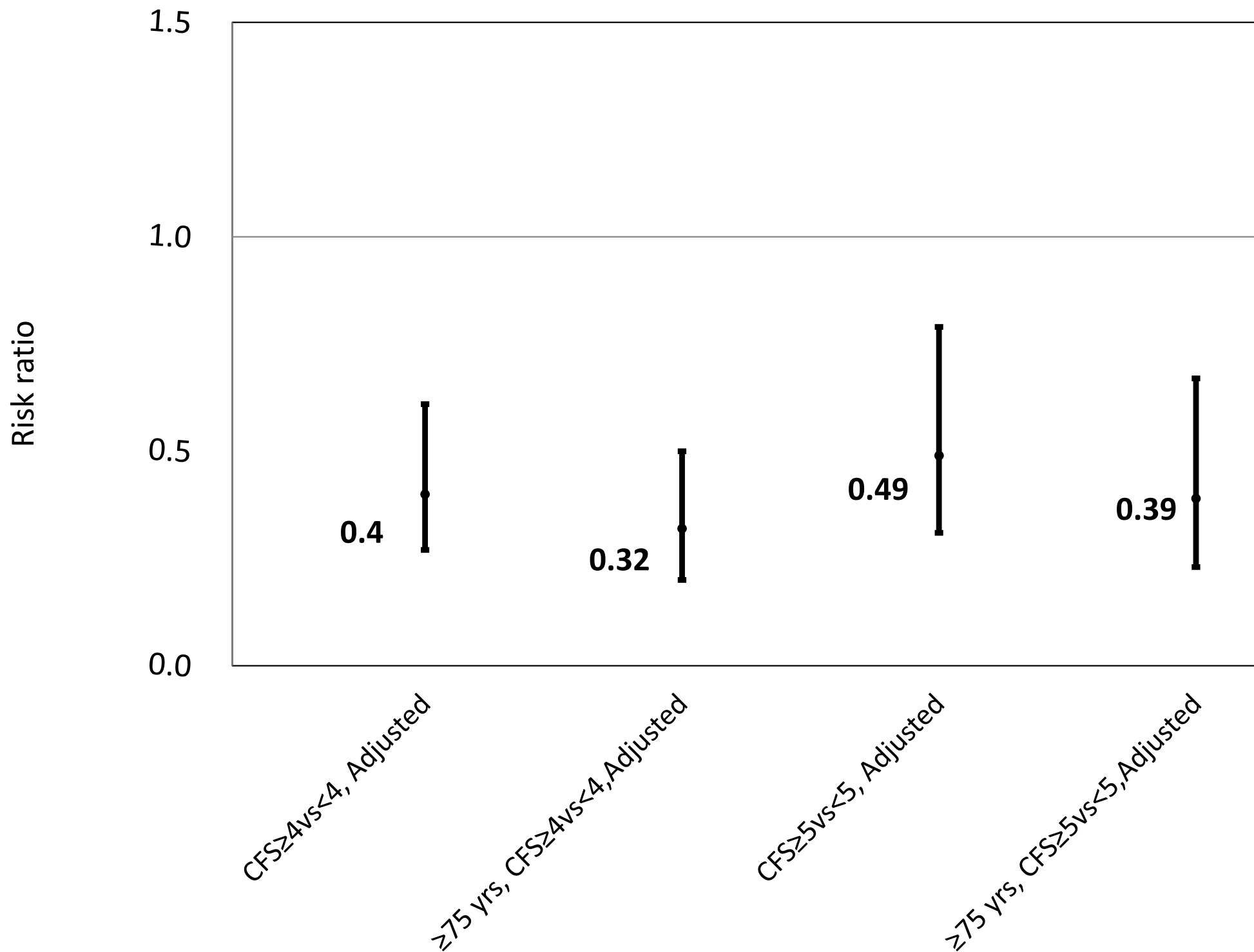

Supplement: Supplementary Material — Supplementary Figure 1. Adjusted risk ratios for tracheostomy according to age group and frailty cutoff. [file 2433-3298-9-3-0643-s001.pdf]
